# *Pseudomonas aeruginosa* isolates from dental unit waterlines can be divided in two distinct groups, including one displaying phenotypes similar to isolates from cystic fibrosis patients

**DOI:** 10.3389/fmicb.2014.00802

**Published:** 2015-01-21

**Authors:** Myriam M. Ouellet, Annie Leduc, Christine Nadeau, Jean Barbeau, Steve J. Charette

**Affiliations:** ^1^Centre de Recherche de l’Institut Universitaire de Cardiologie et de Pneumologie de QuébecQué, QC, Canada; ^2^Institut de Biologie Intégrative et des Systèmes, UniversitéLaval, Québec, QC, Canada; ^3^Département de Biochimie, de Microbiologie et de Bio-Informatique, Faculté des Sciences et de Génie, UniversitéLaval, Québec, QC, Canada; ^4^Faculté de Médecine Dentaire, Université de MontréalMontréal, QC, Canada; ^5^Faculté de Médecine Dentaire, UniversitéLaval, Québec, QC, Canada

**Keywords:** *Pseudomonas aeruginosa*, cluster, RAPD, elastase, biofilm, *Dictyostelium discoideum*, cell lysis

## Abstract

*Pseudomonas aeruginosa* displays broad genetic diversity, giving it an astonishing capacity to adapt to a variety of environments and to infect a wide range of hosts. While many *P. aeruginosa* isolates of various origins have been analyzed, isolates from cystic fibrosis (CF) patients have received the most attention. Less is known about the genetic and phenotypic diversity of *P. aeruginosa* isolates that colonize other environments where flourishing biofilms can be found. In the present study, 29 *P. aeruginosa* isolates from dental unit waterlines and CF patients were collected and their genetic and phenotypes profiles were compared to determine whether environmental and clinical isolates are related. The isolates were first classified using the random amplified polymorphic DNA method. This made it possible to distribute the isolates into one clinical cluster and two environmental clusters. The isolates in the environmental cluster that were genetically closer to the clinical cluster also displayed phenotypes similar to the clinical isolates. The isolates from the second environmental cluster displayed opposite phenotypes, particularly an increased capacity to form biofilms. The isolates in this cluster were also the only ones harboring genes that encoded specific epimerases involved in the synthesis of lipopolysaccharides, which could explain their increased ability to form biofilms. In conclusion, the isolates from the dental unit waterlines could be distributed into two clusters, with some of the environmental isolates resembled the clinical isolates.

## INTRODUCTION

*Pseudomonas aeruginosa* is the most common cause of chronic lung infections affecting individuals with cystic fibrosis (CF). CF is a multi-systemic disease, but the decline in respiratory function is the greatest cause of morbidity and mortality. Individuals suffering from CF produce excessive amounts of mucus in their lungs, which promotes the establishment of opportunistic pathogens that take advantage of a failing pulmonary physiology to establish chronic infections (Murray et al., 2007). Over 80% of CF patients at the age of 30 suffer from *P. aeruginosa* infections (Harrison, 2007). Once installed in the lungs, *P. aeruginosa* changes its behavior to adapt to its new environment by accumulating mutations and rearrangements in its genome (Kresse et al., 2003; Smith et al., 2006). The bacteria form a biofilm in the thick layer of abnormal mucus in the lungs of individuals with CF (Singh et al., 2000), enhancing the resistance of the bacteria to antibiotics and making their elimination by the immune system difficult or impossible (Murray et al., 2007).

*Pseudomonas aeruginosa* harbors a large genome (∼6.5 Mb), and recent studies based on next generation sequencing (NGS) have revealed that there are variations between the genomes of clinical strains, confirming that this species can evolve and adapt during infections (Chung et al., 2012; Rau et al., 2012; Dettman et al., 2013; Marvig et al., 2013; Jeukens et al., 2014). Although NGS is a powerful approach, other more accessible genotyping methods can be used to classify *P. aeruginosa* isolates, including the random amplified polymorphic DNA (RAPD) technique, which has proven particularly useful in epidemiological studies involving *P. aeruginosa* isolates from CF patients (Mereghetti et al., 1998; Ortiz-Herrera et al., 2004; Fothergill et al., 2010b; Hafiane and Ravaoarinoro, 2011). RAPD generates reproducible and often distinctive sets of DNA fragments by subjecting genomic DNA to PCR primed by a single short oligonucleotide of arbitrary sequence. It is a rapid, cost-effective method that requires no prior genetic information on the target organism and that relies on low stringency priming sites for one or more arbitrarily chosen oligonucleotide primers on both strands of the DNA molecule.

*Pseudomonas aeruginosa* is an opportunistic pathogen that is found ubiquitously in water, soil, and plants. It can be also isolated from 2.9 to 50% of water samples collected from dental unit waterlines and from saliva ejector tubing (Barbeau et al., 1996, 1998). There is some evidence that dental unit waterlines colonized with bacteria, including* P. aeruginosa*, may be associated with infections (Martin, 1987; O’Donnell et al., 2011). Because of this, it is vital to identify the genetic and phenotypic features associated with CF infectious isolates from CF patients and to compare them to environmental isolates, particularly those from dental unit waterlines. Many phenotypic and genotypic studies of *P. aeruginosa* isolates have been reported (Deligianni et al., 2010; Fothergill et al., 2010a; Stehling et al., 2010; Bradbury et al., 2011; Hafiane and Ravaoarinoro, 2011; Lelong et al., 2011; Mowat et al., 2011; Chung et al., 2012; Hu et al., 2013; Workentine et al., 2013; Jeukens et al., 2014). However, most of these studies have focused on CF isolates without comparing them to environmental isolates or have compared CF isolates to environmental isolates with respect to a single aspect, such as the type three secretion system or the capacity of the bacteria to resist to predation by *Dictyostelium discoideum* amoebae (Bradbury et al., 2011; Hu et al., 2013). To our knowledge, no broad-based studies comparing the phenotypic and genotypic features of *P. aeruginosa* isolates from CF patients with those of isolates from dental unit waterlines have been conducted.

In the present study, we performed an RAPD analysis of *P. aeruginosa* isolates from dental unit waterlines and from young CF patients with lung infections. Our genotypic analysis enabled us to separate the isolates into three clusters. We also measured various phenotypic traits, including biofilm formation and resistance to amoeba predation to determine how these characteristics correlated with the RAPD clusters. While the clinical and environmental isolates were genetically distinct, some of the environmental isolates displayed the same array of phenotypes as the clinical isolates. However, the main outcome of our study was that there were two genetically and phenotypically distinct groups in the environmental isolates even though they all came from the same environment.

## MATERIALS AND METHODS

### BACTERIA, AMOEBAE, AND CULTURE CONDITIONS

Sixteen clinical and 13 environmental *P. aeruginosa* isolates were studied. The clinical isolates were collected from young CF patients aged 4 to 18 years of age with lung infections (**Table 1**). The environmental isolates were collected from dental unit waterlines at the Faculty of Dentistry of Université de Montréal. **Table 2** lists the names and origins of the environmental isolates. API 20NE tests (BioMérieux) were used to confirm the identity of the *P. aeruginosa* isolates.

**Table 1:** Clinical *Pseudomonas aeruginosa* isolates from cystic fibrosis (CF) patients.

Isolate number	Gender	Birth date	City^a^	Sample date
279	Female	09-04-69	Montréal-Nord	17-05-88
297	Female	25-08-80	Saint-Pie-de-Bagot	01-06-88
358	Female	28-11-80	La Prairie	04-10-88
359	Female	10-03-75	Répentigny	04-10-88
392	Male	16-11-84	Saint-Roch-de-l’Achigan	24-11-88
403	Female	19-04-74	Trois-Rivières	10-01-89
E-500	Female	24-02-76	Mont-Laurier	26-09-89
506	Female	26-08-75	Saint-Blaise	10-10-89
578-A	Male	25-09-73	Montréal-Nord	31-06-90
578-B	Female	15-06-75	Mont-Laurier	31-06-90
585	Female	18-07-72	Saint-Jean sur Richelieu	21-08-90
VD 171	Female	04-11-75	McMasterville	27-10-87
VD 329	Female	04-11-75	McMasterville	23-08-88
VD 706	Female	04-11-75	McMasterville	17-10-89
VD 564	Female	04-11-75	McMasterville	15-05-90
VD 609	Female	04-11-75	McMasterville	05-12-90

*^a^The patients were all from cities in the Province of Quebec, Canada*.

**Table 2:** *P. aeruginosa* isolates from dental unit waterlines.

Dental clinic section	Section abbreviation	Dental unit number
Partial/fixed prosthesis	PPF	1
		2
		7
		13
		18
		19
		20
		21
Emergency	Urg	5
		7
Orthodontics	Ortho	1
Surgery	Chir	D-144
		D-144 assistant

The *P. aeruginosa* isolates were cultivated in LB broth (10 g/L of peptone from casein, 5 g/L of yeast extract, and 10 g/L of NaCl in distilled water) or on LB agar (LB broth supplemented with 12 g/L of Bacto agar) at 37°C. When grown in broth medium, the cultures were shaken at 200 rpm.

We used the DH1-10 strain of *D. discoideum* (Cornillon et al., 2000). The cells were routinely cultivated in HL5 medium [14.3 g/L of Bacto peptone (Oxoid L37), 7.15 g/L of yeast extract, 18 g/L of D-(+)-monohydrate maltose, 0.641 g/L of Na_2_HPO_4_⋅ 2H_2_O, and 0.490 g/L of KH_2_PO_4_ in distilled water with a final pH of 6.5; Froquet et al., 2009] supplemented with 15 μg/mL of tetracycline. The amoebal cultures were grown at 21°C in 10- or 15-cm-diameter polystyrene Petri dishes. The cultures were diluted twice a week in fresh medium to avoid confluence.

### RAPD

Genomic DNA was extracted using a standard procedure (Smith et al., 1989; Wilson, 2001), with an additional RNase treatment (Schmidt et al., 1996), and was optimized for *P. aeruginosa* strains by including an additional 10-min incubation period at 100°C to help lyse the cells at the beginning of DNA extraction. The quality of the genomic DNA was verified by 1% agarose gel electrophoresis in TBE (Tris-Borate-EDTA) buffer. Gels were stained with ethidium bromide and were camera-captured under ultraviolet light. The concentration of genomic DNA was calculated by measuring the optical density at 260 nm, and the quality of the DNA was estimated using the 260 nm/280 nm ratio and gel electrophoresis.

To select suitable candidate primers, 54 random sequence nanomer and decamer oligonucleotides were initially screened using two dental unit isolates and two clinical isolates (data not shown). Primers that generated a low number of distinct bands and at least two polymorphic DNA patterns with these isolates were then tested using the full panel of 29 *P. aeruginosa* isolates. Three primers were selected: 910-07 (5′ -CCGCGGGAG-3′ ), 910-25 (5′ -GCCCGGCAG-3′ ), and OPA-10 (5′ -GTGATCGCAG-3′ ).

Random amplified polymorphic DNA amplifications were performed in 25 μl volumes. The amplification reaction mixtures contained 200 mM each of dATP, dCTP, dGTP, and dTTP (Invitrogen), 1.2 μM primer, 10 or 1 ng of template DNA, and 2.5 units of Taq DNA polymerase (Perkin Elmer) in 10X Amplitaq Gold buffer (Perkin Elmer; 100 mM Tris-HCl, pH 8.3, 500 mM KCl, 15 mM MgCl_2_, and 0.01% gelatin) supplemented with 0.1% (v/v) Triton X-100. A negative control without template DNA was included in each experiment. The reaction mixture was overlaid with sterile mineral oil (Sigma–Aldrich) and was amplified in a DNA Thermal Cycler (Cetus; Perkin Elmer) programmed for one 7-min denaturing pre-cycle at 94°C followed by 30 cycles of 1 min at 94°C, 1 min at 32°C, and 1 min at 72°C followed by a final 7-min elongation step at 72°C. The amplification products, which made up the RAPD fingerprints, were analyzed by the electrophoresis of 10 μl samples on 1.5% agarose gels run in TBE buffer and then stained with ethidium bromide. A 1-kb Plus DNA ladder molecular weight standard (Invitrogen) was included in each gel. The gels were camera-captured under ultraviolet light.

### ANALYSIS OF ISOLATE RELATEDNESS

Computer-assisted analysis of the fingerprints was performed using the Taxotron package (Institut Pasteur), a set of programs for molecular systematics (RestrictoScan®, RestrictoTyper®, Adanson®, and Dendrograph®). The derived molecular sizes (in base pairs) were used to compute distances (D) based on band sharing as the complement of the Dice coefficient: *D* = 1 - (2n_xy_/n_x_ + n_y_), where n_xy_ = the number of bands shared by isolates x and y, and n_x_ and n_y_ = the number of bands scored for each individual. In the pairwise comparison to match co-migrating fragment positions between pairs of RAPD fingerprints, a match was recorded if the normalized molecular size of the first amplicon was within a ±3% window of the molecular size of the second amplicon. A matrix combining all the distances was generated, and a cluster analysis was performed using the unweighted pair-group method with mathematical averages. A dendrogram was then constructed. To generate a dendrogram based on the combined fingerprints obtained with all three primers, the information contained in the three band data files was combined in a new band data file, which was then processed as described above.

### SLIME PRODUCTION

We evaluated slime production as already described (Podbielska et al., 2010). Briefly, we inoculated Congo Red Agar (Brain Heart Infusion, BD supplemented with 5% (w/v) filter-sterilized sucrose, 0.08% (w/v) Congo Red) directly with frozen isolate stocks. The plates were incubated at 37°C for 24 h and then at room temperature for 48 h. We noted the morphologies and characteristics of each isolate and used PA01 and PA14 as controls. Colonies with a mucoid phenotype were scored ++, those with a slightly shiny phenotype were scored +, and flat colonies were scored 0.

### BIOFILM FORMATION

Frozen stocks were inoculated on Tryptone soy agar (BD) plates supplemented with 5% (v/v) sheep blood (Oxoid), which were incubated at 37°C for 24 h. Tryptic soy broth (BD) cultures were inoculated with the precultures and incubated overnight at 37°C without shaking.

The optical densities at 600 nm of the cultures were measured. The cultures were centrifuged, and the pellets were washed twice with potassium phosphate buffer (PBS, 50 mM, pH 7.2) containing 150 mM NaCl. The pellets were suspended in 1.5% (w/v) proteose-peptone (BD) in PBS at an optical density of 0.15 at 600 nm. The suspensions were serially diluted (10^-4^) in the following autoclaved culture medium to measure biofilm formation (in L): 17 g of Tryticase peptone (BBL), 3 g of yeast extract (BBL), 5 g of NaCl (Fisher Scientific), and 2.5 g of sodium phosphate monobasic monohydrate (Acros Organics) to which 0.5% (w/v) filter-sterilized glucose was added. Aliquots (100 μL) of the diluted bacterial suspension were placed in 96-well microplates (16 wells for each dilution; Corning, Costar). The microplates were incubated at 30°C without shaking for 24 h. Uninoculated medium was used as a negative control. The optical density at 620 nm was measured using a microplate reader (AD340; Beckman Coulter). The wells were then washed twice with sterile 0.85% (w/v) NaCl, dried, stained for 15 min with gram crystal violet (BD), rinsed under tap water, and dried again. The stain was decolorized using 200 μL of the following destaining solution: 15% (v/v) acetic acid and 10% (v/v) methanol. The optical density at 570 nm was measured using a microplate reader. Optical densities at 620 nm under 0.1 were considered as no growth. The results are expressed as optical densities at 570 nm using a 10^-4^ inoculum.

### ELASTASE ASSAY

The isolates were grown for 24 h in LB at 37°C with shaking, and the optical densities at 600 nm were measured (Spectronic Biomate 3; Thermo Electron). The cultures were centrifuged, and the elastase activities in the culture supernatants were determined using a previously published protocol (Rust et al., 1994). In brief, 10 mg of elastin-Congo red (Sigma–Aldrich) was added to 1 ml of concentrated culture supernatant diluted with 2 ml of the following buffer: 30 mM Tris, pH 7.2, 1 mM CaCl_2_. The mixtures were incubated for 5 h at 37°C with shaking. The results are expressed as the 495 nm/600 nm ratio.

### PREDATION ASSAY

The assay was used to determine the resistance of the isolates to amoebal predation (Filion and Charette, 2014). The assay was performed in triplicate with each isolate. The bacterial precultures in LB broth in snapcap tubes were incubated overnight at 37°C with a shaking at 200 rpm. The precultures (300 μL) were then gently spread on 10-cm Petri dishes containing 35 ml of autoclaved SM 1/10 agar medium (1 g/L of bactopeptone, 0.1 g/L of yeast extract, 0.22 g/L of KH_2_PO_4_, 0.1 g/L of K_2_HPO_4_, 0.1 g/L of MgSO_4_⋅ 7H_2_O, and 20 g/L of Bacto agar in 950 mL of distilled water supplemented with 50 ml of 2 g/100 mL of filter-sterilized glucose; Filion and Charette, 2014) to obtain uniform bacterial lawns. The Petri dishes were then dried in a laminar flow hood and various concentrations of DH1-10 amoeba from cultures with no more than 60% confluence were suspended in HL5 medium and 5-μL drops were deposited on the bacterial lawns. The concentrations of the amoebae ranged from 0 to 50,000 cells/5 μL. Once the drops had dried, the Petri dishes were incubated at 21°C and were examined for phagocytic plaques after 7 days.

### LYSIS ASSAY

The lysis assay was used to determine whether the *P. aeruginosa* isolates secreted products that can lyse *D. discoideum* cells (Cosson et al., 2002). The lysis assay was performed in sterile polystyrene 24-well plates. HL5 medium (500 μL) containing 500,000 *D. discoideum* cells from cultures at no more than 60% confluence was placed in each well, and the plates were incubated for 1 h at 21°C.

The day before the experiments, bacterial precultures of each *P. aeruginosa* isolate were incubated in 2 mL of LB broth for 24 h at 37°C with shaking at 200 rpm. The bacterial precultures were then centrifuged at 3220 g for 10 min and the supernatants were collected and filter sterilized using 0.20-μm polyethersulfone filters attached to sterile syringes. The HL5 medium in the wells of the polystyrene 24-well plates was gently removed and was carefully replaced by filtered supernatants from the *P. aeruginosa* isolates. The wells were photo-captured at 0, 5, and 10 min using a Moticam Pro 252A camera and Motic Images Advanced 3.2 software (Motic). The procedure was repeated for every supernatant from four different experiments. The disappearance of amoebal refringence is associated with lysis (Cosson et al., 2002). The percentage of cell lysis was determined after a 10 min exposure to the bacterial supernatants. The PAO1 strain was used as a positive control (Cosson et al., 2002).

### CLONING THE 600-bp BAND SPECIFIC FOR CLUSTER III ISOLATES

Random amplified polymorphic DNA using primer OPA-10 was performed on PPF 2 and PPF 7 (cluster III) with parameters identical to those described above, with the following differences. Taq polymerase and standard buffer (M0273L; NEB) were used. No mineral oil was added to the PCR tubes. Hot start PCRs were performed using the following protocol: 94°C when adding the enzyme, followed by 30 cycles of 1 min at 94°C, 1 min at 29°C, 1 min at 72°C, and a final 7-min elongation step at 72°C. The resulting 600-bp RAPD amplification product (OPA-10) was cloned into plasmid pCR^TM^-Blunt II TOPO®(Invitrogen) according to manufacturer’s protocol.

Insert sequencing was performed at the IRIC Genomic Platform (Université de Montréal). A blast analysis (see Results section for details) revealed that the 600-bp fragment corresponds to a region overlapping the *wbjC* and *wbjD* genes, both of which code for epimerases.

### Gyrase B AND *wbjC*–*wbjD* PCR GENOTYPING

One primer pair was designed for the gyrase B gene used as a positive PCR control (GyrB-F1: 5′ -GAG TAC CTG AAC ACC AAC AAG A-3′ + GyrB-R1: 5′ -AGT CAC CCT CCA CGA TGT A-3′ ). It generated a 604-bp amplicon. Another primer pair was designed to target a region overlapping the *wbjC* and* wbjD* genes (EPI-F2: 5′ -CCT GGA TGG ACT CAT GAC ATT AC-3′ + EPI-R2: 5′ -CAT GAC CCT AGA CAA GCG AAT AA-3′ ). It generated a 212-bp amplicon. The primers were synthesized by Life Technologies.

Either purified bacterial DNA (10 ng, see the RAPD section) or 3 μl of diluted cell lysates (1:10) from single colonies suspended in 20 μl of SWL buffer [50 mM KCl, 2.5 mM MgCl_2_, 10 mM Tris, pH 8.3, and 0.45% (v/v) NP-40 and Tween 20] were used for the PCR templates depending on the availability of the material. In the case of the lysates, samples were heated at 95°C for 5 min in a thermocycler to complete the lysis.

The conditions described for the RAPD procedure were used except that the gelatin and Triton X-100 were omitted. The hot start protocol was as follows: an initial 2.5-min incubation at 95°C followed by 30 cycles of 30 s at 95°C, 30 s at 55°C, and 1 min at 72°C, followed by a final 7-min elongation step at 72°C. The PCR was performed twice for each isolate.

## RESULTS

### THE RAPD ASSAY SEPARATED THE *P. aeruginosa* ISOLATES INTO THREE CLUSTERS

**FIGURE 1:**
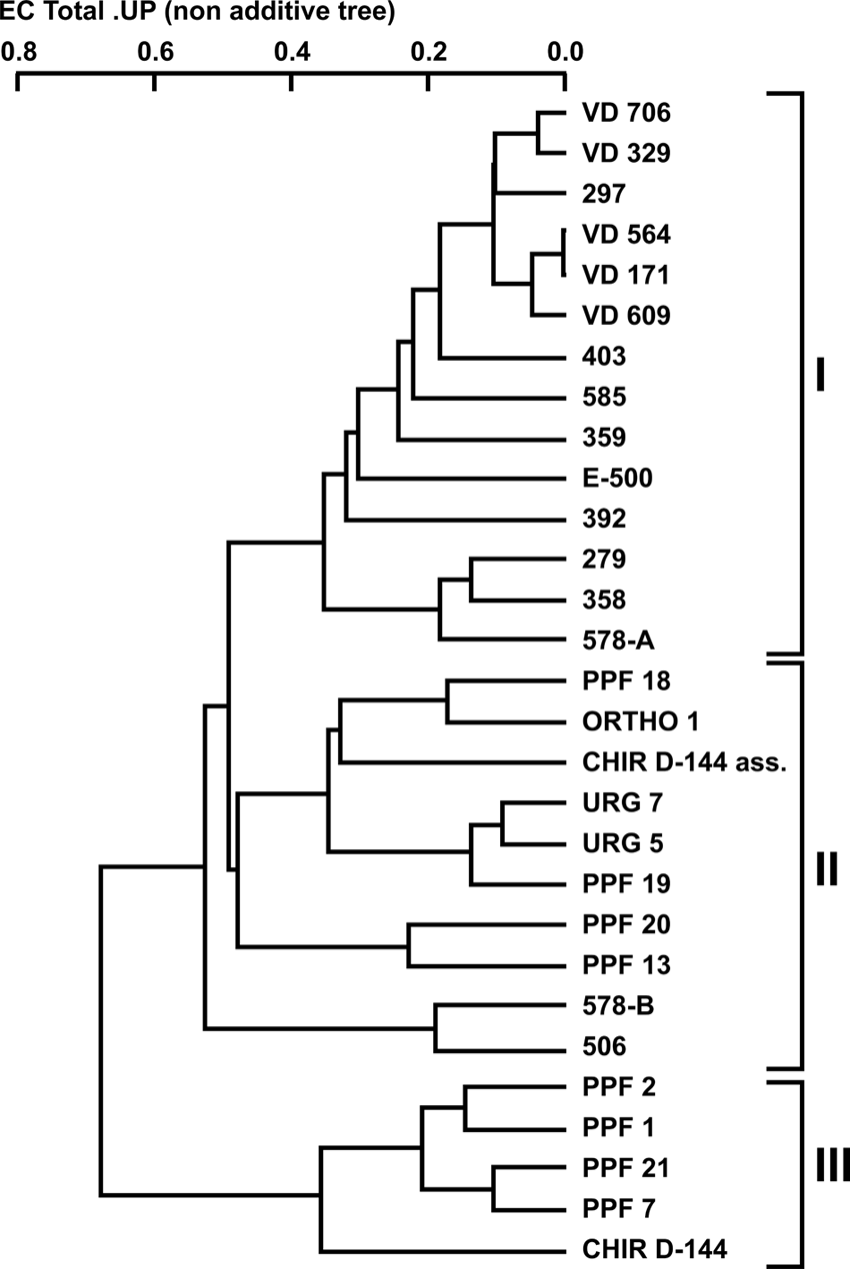
**Dendrogram of the combined random amplified polymorphic DNA (RAPD) fingerprints of *Pseudomonas aeruginosa* clinical and dental unit waterline isolates using the 910-25, 910-07, and OPA-10 primers**.

Twenty-nine *P. aeruginosa* isolates [16 from CF patients (clinical) and 13 from dental unit waterlines (environmental)] were collected and compared (**Tables 1** and **2**). Three random probes were chosen for the RAPD assay in order to genotype the isolates and generate a dendrogram (see Materials and Methods). The RAPD assay easily discriminated the *P. aeruginosa* isolates into clinical and environmental clusters (**Figure 1**). Three clusters (clusters I, II, and III) were generated using a genetic distance of 60% as a threshold. At this similarity level, both the clinical and environmental isolates clearly clustered into distinct groups. The intracluster similarity for each cluster was approximately 65% or greater.

All the clinical isolates belonged to cluster I, except for isolates 506 and 578-B, which belonged to cluster II. The VD series *P. aeruginosa* isolates, which were isolated from the same patient over a 3-year period, shared 90% similarity. Isolate 297, which was isolated from an unrelated patient, shared strong genetic similarity (90%) with the VD series isolates. The environmental isolates were placed in clusters II and III even though they were all isolated in the same building and, as such, from the same municipal water supply. No environmental isolates were included in cluster I. Based on this clear genetic segregation of the isolates, we hypothesized that some phenotypic characteristics associated with virulence might also be distinctively associated with the three clusters.

### PHENOTYPES ASSOCIATED WITH THE CLUSTERS

The aspects of colony isolates were recorded. Three different phenotypes were observed. Colonies of some isolates had a mucoid phenotype (**Table 3**). These isolates were only found in cluster I (clinical isolates) but not all the clinical isolates presented a mucoid phenotype. Some isolates of cluster I had a slightly shiny phenotype, which was also observed with most isolates in cluster II. Lastly, all the isolates in cluster III had a flat colony phenotype.

**Table 3:** Colony appearance of the *P. aeruginosa* isolates.

*P. aeruginosa* isolate	Cluster	Colony phenotype
VD 706	I	++
VD 329	I	++
297	I	+
VD 564	I	+
VD 171	I	++
VD 609	I	++
403	I	+
585	I	+
359	I	++
E-500	I	0
392	I	+
279	I	++
358	I	0
578-A	I	++
PPF 18	II	+
Ortho 1	II	+
Chir D-144 assistant	II	+
Urg 7	II	+
Urg 5	II	+
PPF 19	II	+
PPF 20	II	+
PPF 13	II	+
578-B	II	0
506	II	+
PPF2	III	0
PPF1	III	0
PPF21	III	0
PPF7	III	0
Chir D-144	III	0

*Colonies with a mucoid phenotype were scored ++, those with a slightly shiny phenotype were scored +, and flat colonies were scored zero*.

**FIGURE 2:**
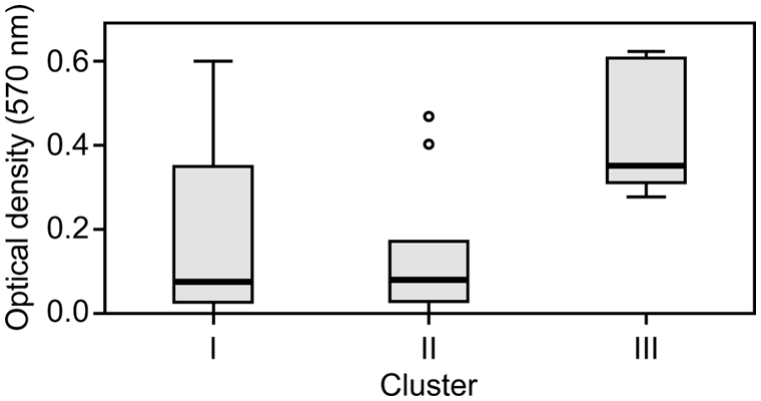
**Boxplot of biofilm-forming capacity.** Isolates in cluster III produced significantly more biofilm than those in clusters I and II. Kruskal–Wallis with independent variables.

**FIGURE 3:**
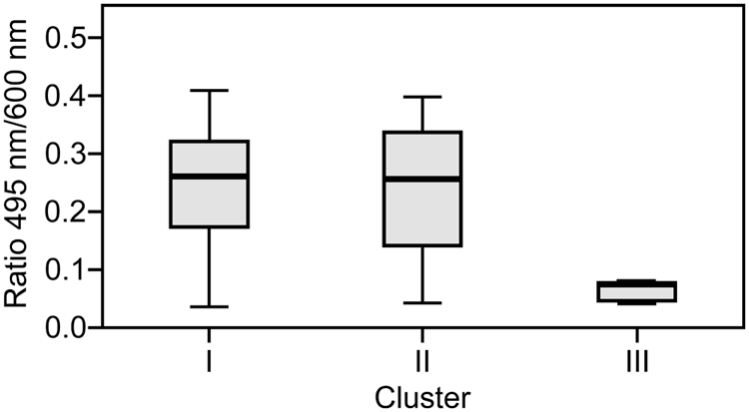
**Boxplot of elastase production.** Isolates in cluster III produced significantly less elastase than those in clusters I and II. Kruskal–Wallis with independent variables.

The capacity to form biofilms was evaluated in polystyrene microplates using a crystal violet assay. When the isolates were compared based solely on their clinical or environmental origin, no significant differences were noted, although the environmental isolates had a slight tendency to produce more biofilm. However, significant differences were observed when the capacity of the isolates in the three clusters to form biofilms was evaluated. Cluster III included isolates that formed significantly more biofilm than those in clusters I (*p* = 0.042) and II (*p* = 0.012; **Figure 2**). Elastase production also discriminated cluster III from clusters I and II (*p* = 0.034; **Figure 3**). No differences were noted between cluster I and II in regards to biofilm formation and elastase activity.

### THE INTERACTION OF *P. aeruginosa* WITH AMOEBAE WAS PARTIALLY ASSOCIATED WITH THE CLUSTERS

**FIGURE 4:**
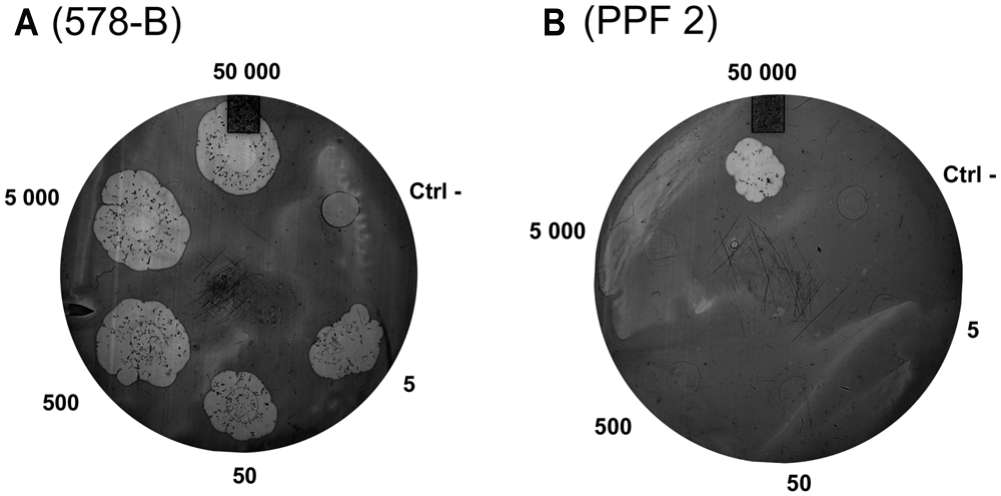
**Example of results obtained with the amoeba predation assay.** Various quantities of amoebae were deposited on *P. aeruginosa* lawns (5 to 50,000 amoebae per spot). After a 7-day incubation, phagocytic plaques were visible where amoebae fed on the bacteria. The growth of the amoebae fed on the lawns of two isolates is illustrated: **(A)** Isolate 578-B is sensitive to predation; **(B)** Isolate PPF 2 is resistant to predation. The negative control (Ctrl-) was HL5 medium without amoeba.

The *D. discoideum* predation assay could not distinguish between the clinical and environmental isolates or the clusters. (**Figure 4**; **Table 4**). Two clinical isolates and one environmental isolate of the 29 tested were not resistant to amoebal predation. All the other isolates were highly resistant to predation. Only the highest concentration of *D. discoideum* cells was able to produce phagocytic plaques in the bacterial lawns of the majority of the isolates. The three most permissive isolates (392, 578-B, and PPF7) allowed the growth of *D. discoideum* when as few as 5 to 50 amoebal cells were deposited on the bacterial lawns. None of the three permissive isolates belonged to a same cluster. Clinical isolates 392 and 578-B belonged to cluster I and II, respectively, and environmental isolate PPF7 belonged to cluster III. There was thus no correlation between the capacity of the *P. aeruginosa* isolates to resist amoebae predation and their RAPD cluster.

**Table 4:** Results of the amoeba predation assay.

*P. aeruginosa* isolate	Cluster	Average^a^
VD 706	I	1.0
VD 329	I	0.7
297	I	1.0
VD 564	I	1.0
VD 171	I	1.0
VD 609	I	1.0
403	I	1.0
585	I	2.0
359	I	0.7
E-500	I	1.3
392	I	5.0
279	I	1.0
358	I	2.3
578-A	I	1.3
PPF 18	II	1.0
Ortho 1	II	1.0
Chir D-144 assistant	II	1.0
Urg 7	II	1.0
Urg 5	II	1.0
PPF 19	II	1.0
PPF 20	II	1.0
PPF 13	II	1.0
578-B	II	5.0
506	II	1.0
PPF2	III	1.0
PPF1	III	1.7
PPF21	III	1.3
PPF7	III	3.7
Chir D-144	III	1.7

*The values are the average of three independent experiments*.

*^a^A value is associated with the number of amoebae required to produce a phagocytic plaque in the bacterial lawn (see **Figure 4**): (1) phagocytic plaques appear with 50,000 amoebae; (2) phagocytic plaques appear with 5,000 amoebae; (3) phagocytic plaques appear with 500 amoebae; (4) phagocytic plaques appear with 50 amoebae; and (5) phagocytic plaques appear with five amoebae. The value is zero when the isolate is totally resistant to predation by the amoebae (i.e., no observable phagocytic plaque)*.

**FIGURE 5:**
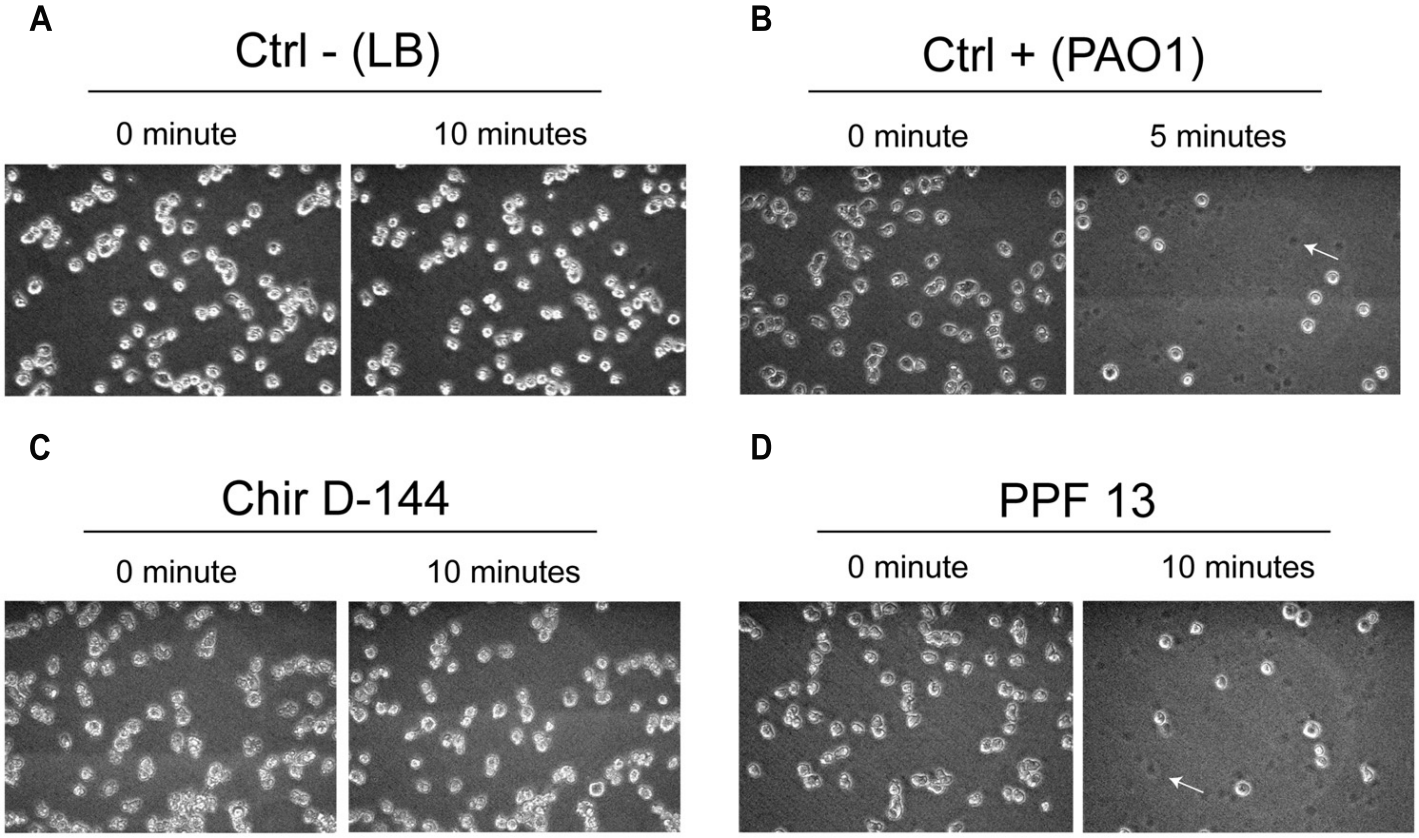
**Lysis of *Dictyostelium discoideum* cells exposed to *P. aeruginosa* culture supernatants.** *P. aeruginosa* culture supernatant in contact with amoebae grown in the wells of 24-well plates. The amoebae were overlain with *P. aeruginosa* culture supernatants, and images were acquired at 0, 5, and 10 min. The images taken at each time correspond to the same field. **(A)** *D. discoideum* cultures overlain with fresh culture medium were used as negative controls. No cell lysis was observed. **(B)** PAO1 culture supernatants were used as positive controls (Cosson et al., 2002). A large number of *D. discoideum* cells were already lysed after 5 min. **(C)** Example of an isolate (Chir D-144) that was unable to lyse amoebae. **(D)** Example of an isolate (PPF 13) that caused marked cell lysis. Dead cells left a dark spot (arrow).

The ability to lyse amoebae differentiated between clinical and environmental isolates and between clusters (**Figure 5**; **Table 5**). Isolates from clusters I and II were either able to lyse amoebal cells or were variable in their ability. While some isolates lysed amoebal cells, others did not and, for some isolates, the ability was highly variable from one experiment to another (**Table 5**). Variability was highest in cluster I. The lysis assay performed on the environmental isolates separated them into two groups: isolates that lysed amoebal cells (cluster II) and isolates that did not (cluster III; **Figure 5**; **Table 5**). In cluster II, the majority of the isolates were able to lyse amoebal cells, with the exception of Ortho1, 506, and urg5, which had a variable lytic phenotype, and 578-B, which displayed no lytic activity. No isolates in cluster III were able to lyse amoebal cells (**Table 5**).

**Table 5:** Lysis of *Dictyostelium discoideum* cells exposed to *P. aeruginosa* culture supernatants.

*P. aeruginosa* isolates	Clusters	Average (% of cells)	SD	Outcome^a^
				
VD 706	I	107.4	1.1	N
VD 329	I	41.1	38.8	V
297	I	62.5	47.3	V
VD 564	I	111.3	22.1	V
VD 171	I	81.7	28.2	V
VD 609	I	100.3	16.0	N
403	I	23.1	44.9	V
585	I	109.8	3.6	N
359	I	91.9	61.2	V
E-500	I	78.2	49.3	V
392	I	5.6	11.2	**L**
279	I	94.9	21.2	V
358	I	62.0	63.6	V
578-A	I	97.5	7.6	N
PPF 18	II	6.1	10.2	**L**
Ortho 1	II	14.0	27.1	V
Chir D-144 assistant	II	0.5	0.7	**L**
Urg 7	II	5.2	10.5	**L**
Urg 5	II	30.1	48.1	V
PPF 19	II	9.5	17.3	**L**
PPF 20	II	6.7	12.4	**L**
PPF 13	II	13.6	8.8	**L**
578-B	II	111.6	2.4	N
506	II	62.8	57.1	V
PPF2	III	107.6	9.2	N
PPF1	III	94.9	12.9	N
PPF21	III	96.0	3.5	N
PPF7	III	113.0	6.7	N
Chir D-144	III	105.2	2.9	N

*The values are the average and SD of four independent experiments*.

*^a^L, lysis; N, no lysis (or no significant lysis); V, variable*.

### GENES ENCODING EPIMERASES SPECIFIC TO CLUSTER III

**FIGURE 6:**
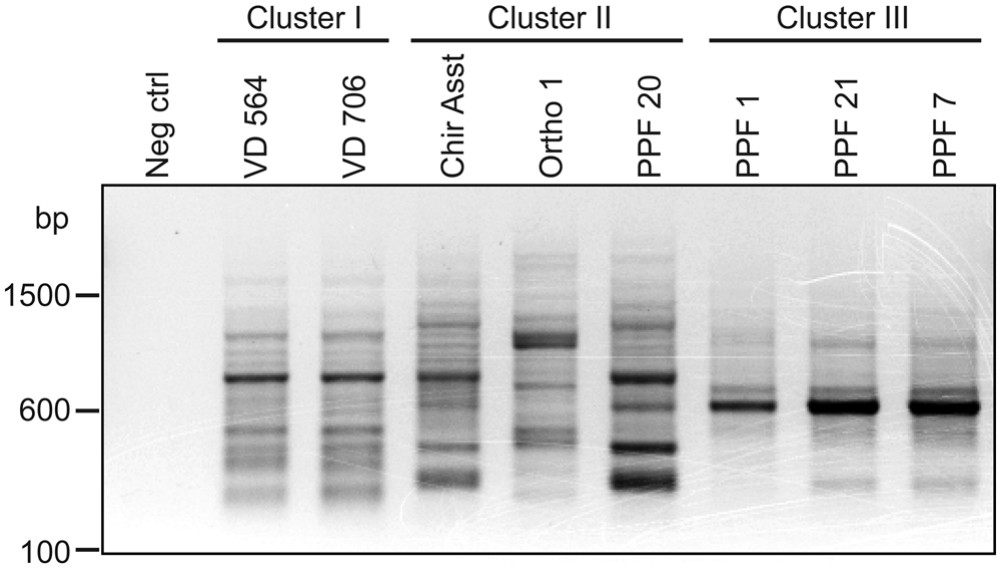
**Agarose gel electrophoresis of RAPD amplification products generated by the OPA-10 primer.** Neg control refers to the negative control (no template DNA).

As illustrated by **Table 6**, which presents a summary of the phenotypic results obtained in this study, it appeared that *P. aeruginosa* isolates from the cluster III are those with the most distinctive phenotypes compared to the two other clusters even if isolates in cluster I displayed important variability for some of the phenotypes analyzed. In RAPD, an intense band with the OPA-10 primer was observed at 600 bp for cluster III isolates (**Figure 6**). With the objective to have molecular hints on the distinctive characteristics of the cluster III, the intense 600-bp band specific to this cluster obtained in RAPD using the OPA-10 primer was purified, cloned in a vector and sequenced to establish the identity of this DNA amplicon. The sequence of the 600-bp band had 99 to 100% identity with a specific genomic region of many *P. aeruginosa* strains (VRFPA04, accession number CP008739.2; B136-33, CP004061.1; NCGM 1984, AP014646.1; NCGM 1900, AP014622.1; NCGM2.S1, AP012280.1). This genomic region overlaps *wbjC* and *wbjD* genes, which encodes an O11 O-antigen biosynthesis associated epimerase and an UDP-N-acetylglucosamine 2-epimerase, respectively. These enzymes are involved in the synthesis of UDP-N-acetyl-L-fucosamine, a precursor involved in the production of lipopolysaccharides (LPS) in *P. aeruginosa* serotype O11 (Kneidinger et al., 2003; Mulrooney et al., 2005).

To confirm that the *wbjC* and *wbjD* genes are specific to *P. aeruginosa* isolates from the cluster III, a PCR-based genotyping test was performed on all the *P. aeruginosa* isolates using a primer pair specific to *wbjC* and *wbjD* genes and another targeting the *gyrB* gene as positive control. While the *gyrB* primer pair gave an amplicon for every isolates, only those of the cluster III allowed a positive PCR signal for the *wbjC* and *wbjD* genes confirming that these genes are only present in isolates of cluster III.

## DISCUSSION

We investigated the genetic and phenotypic characteristics of environmental isolates of* P. aeruginosa* to determine their similarity with clinical isolates. This was the first time that a large number of environmental isolates from dental unit waterlines were compared using RAPD and multiple phenotypic tests, including an amoeba predation assay. To determine whether there was a marked evolution of the bacteria in the lungs of CF patients as previously proposed (Kresse et al., 2003; Smith et al., 2006), we also analyzed clinical isolates from young CF patients between 4 and 18 years of age. We anticipated that the CF isolates would be genetically and phenotypically similar to the bacteria that had initially infected these patients.

We used RAPD to classify the *P. aeruginosa* isolates into clusters. We used three primers generating amplification patterns with a strong discriminatory potential. All the clinical isolates except two could be grouped into one cluster (cluster I). Since all the CF patients in the present study were treated in the same clinic, it was assumed that they would have harbored a limited number of strains, including variants, during the sampling period. On the other hand, we looked for relatively common, stable genetic characteristics in clinical isolates. The marked genetic similarity among the *P. aeruginosa* isolates collected over a 3-year period from the same patient (VD isolates; **Table 1**) showed that the isolates were relatively genetically stable. However, subtle changes (point mutations) in the genome of the isolates that can occur over a 3-year period might not have been detected by RAPD.

The clinical strains displayed greater variability than the environmental isolates in the phenotypic assays. Phenotypic variability of this kind has been reported previously (Deligianni et al., 2010). *P. aeruginosa* strains in CF lungs are subject to considerable selection pressure, which can increase the need of the strains to adapt through increased mutation rates, a phenomenon called the insurance effect (Boles et al., 2004).

A striking feature of our RAPD analysis was that environmental isolates could be segregated into two clusters. Interestingly, these two clusters displayed different phenotypic characteristics. **Table 6** presents a summary of the phenotypic trends observed in each cluster. The environmental isolates in cluster III displayed divergent and even opposite phenotypes compared to the isolates in the other two clusters. This is especially remarkable for the mucoid, biofilm formation, and capacity to lyse amoebae phenotypes. The environmental isolates in cluster II displayed phenotypes that were more similar to the clinical isolates. Genetic similarities based on RAPD results between clusters I and II could explain the phenotypes observed. In this regard, all the phenotypic differences observed between the cluster III and other isolates were in agreement with those reported in previous publications (Rocchetta et al., 1999; Smith et al., 2006). For example, the mucoid clinical *P. aeruginosa* strains from a patient with a chronic pulmonary infection, had a lower capacity to produce an adherent biofilm, and were less virulent. It appears that virulence factors are recognized by the immune system and that bacteria presenting virulence factors are eliminated. B-band LPS, which are the predominant polysaccharides of non-mucoid strains such as cluster III isolates, are anionic and may play a key role in binding and may thus promote the formation of a more adherent biofilm, as was observed with cluster III isolates.

**Table 6:** Phenotypic trends of the three clusters^**a**^.

Cluster	Mucoid phenotype	Biofilm production	Elastase activity	Predation resistance	Amoeba lysis	Epimerase genes
I	++ (variable)	+ (variable)	+++	+++	Highly variable	-
II	+	+	+++	+++	++	-
III	-	+++	+	+++	-	+

*^a^Marked phenotypic variability was observed among cluster I isolates. The presence and intensity of a phenotype is indicated by “-” (not present) to “+++” (very intense)*.

Our analysis revealed that the dental unit waterlines contained two distinct populations of *P. aeruginosa*. It has been previously shown that dental unit waterlines are colonized by biofilms where opportunistic waterborne pathogens can proliferate (Barbeau et al., 1997; Walker et al., 2000). Earlier studies at Université de Montréal have shown that* P. aeruginosa* has a non-random distribution in these dental units since 89.5% of the isolates were isolated from only four of the nine clinics at the Faculty of Dentistry (Barbeau et al., 1996). There was no physical link between the dental units apart from the fact that they were all connected to the same water source (municipal drinking water). It was not possible to establish an association between a given dental unit and a given cluster. Since the environmental isolates from cluster III formed abundant biofilms, without being mucoid, these isolates may have originated from biofilms in the dental unit waterlines. The environmental isolates in cluster II, which did not form substantial biofilms *in vitro*, may have come from planktonic populations, which were transiently present in the dental unit waterlines.

The segregation of the environmental isolates into two distinct clusters revealed a number of interesting features. Strains in cluster III produced significantly more biofilm, displayed less elastase activity, and were unable to lyse amoebal cells despite the fact that their resistance to predation was not significantly different from that of the isolates in clusters I and II. This suggested that other factors that were not tested in the present study are involved in the interaction between *P. aeruginosa* and *D. discoideum*.

The predation resistance of the *P. aeruginosa* isolates of various origins has been tested using *D. discoideum* as the predator in four previous studies (Bradbury et al., 2011; Lelong et al., 2011; Janjua et al., 2012; Jeukens et al., 2014). *P. aeruginosa* strains isolated from CF patients of all ages (babies to adults) have been studied, including isolates that can spread to other patients like the Liverpool epidemic strain (LES). Isolates from urinary tract infections, skin ulcers, intubated patients, and the environment have also been tested using the *D. discoideum* predation assay. Even though the studies used different versions of the predation assay in which the amoeba strains, media, or scoring parameters were different, general conclusions can be drawn. Most of the *P. aeruginosa* isolates tested were resistant to *D. discoideum* predation. Half the isolates from CF teenagers, most isolates from CF adults, and all the transmissible CF isolates displayed little or no resistance to amoeba predation. Some authors suggested that the virulence of *P. aeruginosa* decreases when it adapts to the CF lung environment over a long period of time (Smith et al., 2006; Friman et al., 2013). In this study, we analyzed *P. aeruginosa* isolates from young CF patients. All the isolates, except two, were highly resistant to amoeba predation and produced high levels of elastase, suggesting that they had not begun the adaptation-associated loss of virulence process. Our environmental isolates were resistant to *D. discoideum* predation, which is in agreement with previously published predation results for other environmental isolates (Bradbury et al., 2011). It thus appears that the predation assay is not the most sensitive assay for discriminating* P. aeruginosa* isolates of various origins. On the other hand, the cell lysing capacity of the isolates was a more useful parameter that made it possible to segregate the dental unit waterline isolates into two clusters based on their interactions with amoebae.

It is important to remember that the predation assay measures the cumulative effect of the entire arsenal deployed by a bacterium to resist predation by amoebae. However, it is possible that different “weapons” in the bacterial arsenal can result in the same level of resistance. For example, the cytolytic potential of cluster I and II isolates and the increased production of biofilms by cluster III isolates are different “weapons,” but are both potentially efficient ways to resist amoeba predation.

Our molecular analyses showed that cluster III isolates are the only ones harboring the *wbjC* and *wbjD* genes, which encode epimerases. As mentioned earlier, these enzymes are required for the synthesis of LPS. To our knowledge, no studies have investigated these epimerases to determine their involvement in biofilm formation. However, the contribution of other epimerases in biofilm formation has been studied in other bacterial species. For example, the *galE* gene that encodes UDP-galactose-4-epimerase is essential for the formation of biofilms by *Xanthomonas campestris* pv. *campestris* and *Vibrio cholerae* (Nesper et al., 2001; Li et al., 2014), while the overexpression of the *galE* gene by *Thermus thermophilus* results in an increase in the production of biofilms (Niou et al., 2009). Surprisingly, the inactivation of the *galE* gene in *Haemophilus parasuis* and *Porphyromonas gingivalis* has the opposite effect, with higher biofilm production in mutant strains than in the wild-type bacteria (Nakao et al., 2006; Zou et al., 2013). In addition, the inactivation of ADP-glycero-manno-heptose 6-epimerase, which is encoded by *GmhD* in *Vibrio vulnificus*, results in a defect in the production of mature LPS, attenuating the ability to form a biofilm (Kim et al., 2007). While their effects are not always clear, epimerases do appear to play a role in biofilm formation.

Of all the isolates analyzed, cluster III isolates displayed the greatest ability to form biofilms. So far, we cannot conclude that the increase in biofilm production is linked to the *wbjC* and *wbjD* genes but this study stresses the need to further invest this finding. Above all, it shows that the RAPD technique is a valuable approach for providing molecular insights into differences between various groups of isolates.

It is also interesting that the *wbjC* and *wbjD* genes are only found in a subset of strains/isolates, both in our study and in various genomic databases. For example, GenBank lists 301 *P. aeruginosa* genome assemblies, 40 of which have the 600-bp sequence specific to cluster III isolates that overlaps the *wbjC* and *wbjD* genes. The origins of the strains/isolates are rudimentarily indicated in the databases for 15 of the 40 genomes. These strains originated from infected corneas, community-acquired diarrhea, blood from a patient with an infection, urine from a patient with a urinary tract infection, sputum, gastric juice from a dolphin kept in captivity, a fuel tank, and a biofilm in an industrial water system. It is important to note that none of the origins are related to CF, further indicating that *P. aeruginosa* isolates harboring the *wbjC* and *wbjD* genes form a distinctive group that is likely not involved in chronic pulmonary infections.

## Conflict of Interest Statement

The authors declare that the research was conducted in the absence of any commercial or financial relationships that could be construed as a potential conflict of interest.
